# Effects of exosome on the activation of CD4+ T cells in rhesus macaques: a potential application for HIV latency reactivation

**DOI:** 10.1038/s41598-017-15961-x

**Published:** 2017-11-15

**Authors:** Xiaowu Hong, Blake Schouest, Huanbin Xu

**Affiliations:** 1 Department of Immunology, School of Basic Medical Sciences, Fudan University, Shanghai, China; 2 Tulane National Primate Research Center, Pathology and Laboratory Medicine, Tulane University School of Medicine, Covington, LA 70433 USA

## Abstract

Exosomes are small extracellular vesicles (EVs), released by a wide variety of cell types, carry donor origin-proteins, cytokines, and nucleic acids, transport these cargos to adjacent or distant specific recipient cells, and thereby regulate gene expression and activation of target cells. In this study, we isolated and identified exosomes in rhesus macaques, and investigated their effects on cell tropism and activation, especially their potential to reactivate HIV latency. The results indicated that plasma-derived exosomes preferentially fuse to TCR-activated T cells and autologous parent cells. Importantly, the uptake of exosomes, derived from IL-2 stimulated CD4+ T cells, effectively promoted reactivation of resting CD4+ T-cell, as indicated by an increased viral transcription rate in these cells. These findings provide premise for the potential application of exosome in the reactivation of HIV latency, in combination its use as functional delivery vehicles with antiretroviral therapy (ART).

## Introduction

Extracellular vesicles (EVs) are membrane-derived lipid bilayer particles that are released by cells in the body. It is believed that most mammalian cells are able to release EVs, which include exosomes, microvesicles, and apoptotic bodies. Small heterogeneous exosomes (30~100 nm) are distinguished from shedding microvesicles (also referred to as ectosomes or lysosomes) or apoptotic bodies that form as a result of direct budding from the plasma membrane, as they are initially produced by a process of interaction with the Golgi complex to form bilayer endosomal membrane multivesicular bodies (MVBs)^1,2^. The markers of exosomes include multiple families of proteins on parent cells, such as tetraspanins (CD63, CD81 and CD9), heat shock proteins (HSP70), and MHC class I and class II molecules^3–7^. The contents of exosomes mostly reflect their cellular origin, including molecules potentially involved in activation. For example, T cell receptors are abundantly present on exosomes secreted by T lymphocytes^8^. Exosomes carry cellular origin-specific proteins, lipids, as well as nucleic acid materials in the form of DNA, mRNA, microRNA (miRNA) and noncoding RNA^9–11^. Exosomes not only transport cargos into the adjacent or distant target cells, even across the blood-brain barrier (BBB) via membrane fusion, endocytosis or receptor-mediated internalization, but also promote cell activation by receptor signaling^12,13^. Therefore, exosomes play important roles in multidirectional crosstalk between cells under normal and pathological conditions^1,14^. Besides engineered exosomes that are used as therapeutic carriers of drugs and miRNAs^15,16^, exosomes are able to induce quiescent cell activation through ADAM17^17,18^, Toll-like receptor (TLR), RNA polymerase II^19^, NF-κB^20^, or trans-activating responses (TAR) element from HIV-infected cells^21,22^. Exosomes from either productively HIV-infected or uninfected cells have been reported to activate HIV latency^17,19^.

Residual low-level replication-competent HIV-1 persists in a latent state in the form of integrated and transcriptionally silent proviruses even after long-term ART, resulting in lifelong infection and viral rebound to pre-treatment levels when ART is discontinued^23–28^. The size of the HIV-1 reservoir differs in tissues, with higher frequency on a per-cell basis in lymph nodes, rectum, spleen and lung^29,30^. Latently infected cells include macrophages and dendritic cells (DC) in blood, GALT and the CNS^31–34^, yet the most abundant and long-term HIV cellular reservoirs are resting memory CD4+ T cells (over 20 years)^35,36^, representing the major hurdle to virus eradication in patients. Since the transcription of HIV genes depends on the activation state of cells, the integrated HIV DNA is transcriptionally silent in resting T cells^37,38^. Thus, the “shock and kill” strategy has been proposed to reverse HIV-1 latency in viral reservoirs by latent stimulators in combination with ART. Cells harboring latent HIV provirus may be activated by IL-2^39,40^, lipopolysaccharides (LPS)^41^, bacterial superantigens^42^, anti-T cell antibodies (OKT3)^43^, or other latency-reversing agents (LRAs) such as Histone deacetylase inhibitors (HDACi) and protein kinase C (PKC) activators. Once reactivated, the virus latently infected cells are more easily eliminated through viral cytopathic effects or host cytolytic T lymphocyte (CTL) responses^44,45^. In this study, we characterized exosomes from rhesus macaques and investigated their effects on uptake by CD4+ T cells and reactivation of HIV latency.

## Results

### Identification of plasma exosomes in rhesus macaques

**Figure 1:**
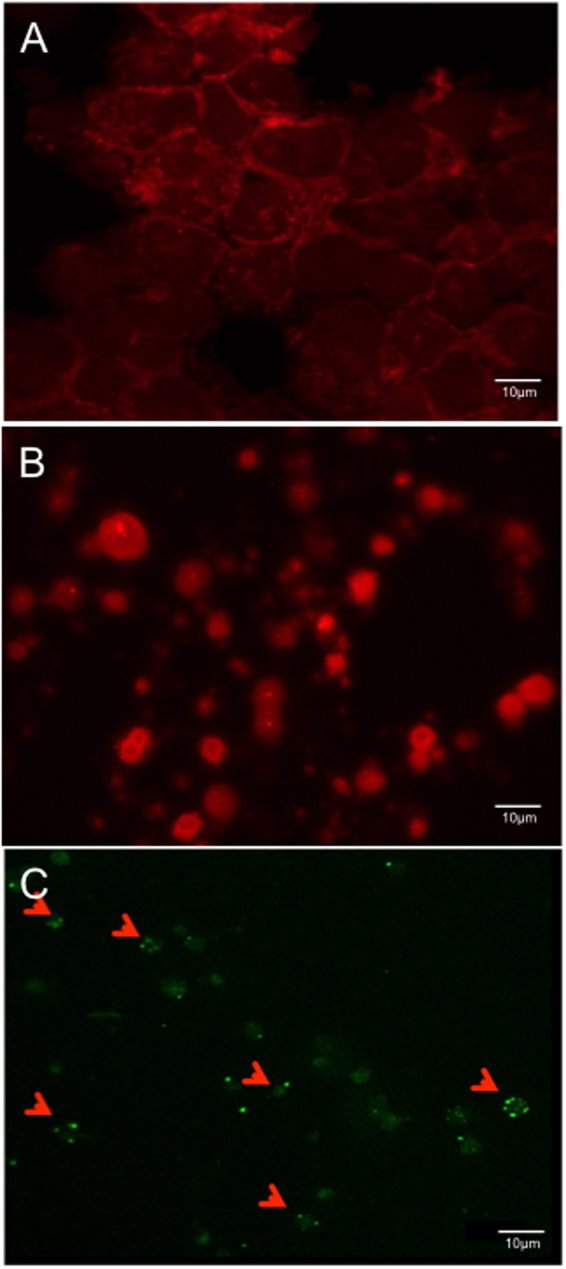
Isolation and identification of plasma exosomes in rhesus macaques. PBMC (**A**) and exosomes (**B**) stained by BODIPY TR, or exosomes stained by CD63 (**C**). Exosomes were isolated from plasma, stained by BODIPY TR, and spun onto the slide. Note CD63 aggregation on the membrane of small exosome vesicles in heterogeneous size.

Exosomes are small membrane vesicles with heterogeneous size and round-shaped morphology, released from most mammalian cells^46^. To isolate the intact exosomes from plasma in rhesus macaques, total exosomes were precipitated in terms of their lower solubility, compared with other isolation techniques^47^. The BODIPY TR Ceramide, a red-fluorescent dye to label lipids in the plasma membrane and Golgi apparatus, was used to efficiently label the membrane of isolated exosomes. As indicated by Fig. 1B, the plasma-derived exosomes from rhesus macaques showed a heterogeneous range of sizes, compared with the more uniform size and morphology of PBMCs (Fig. 1A). Consistent with previous reports^48,49^, exosomes displayed transmembrane CD63, which aggregated around exosome vesicles (Fig. 1C). These results demonstrated that heterogeneous exosomes from the plasma of rhesus macaques could be successfully isolated by precipitation.

### Cellular tropism of plasma exosomes

**Figure 2:**
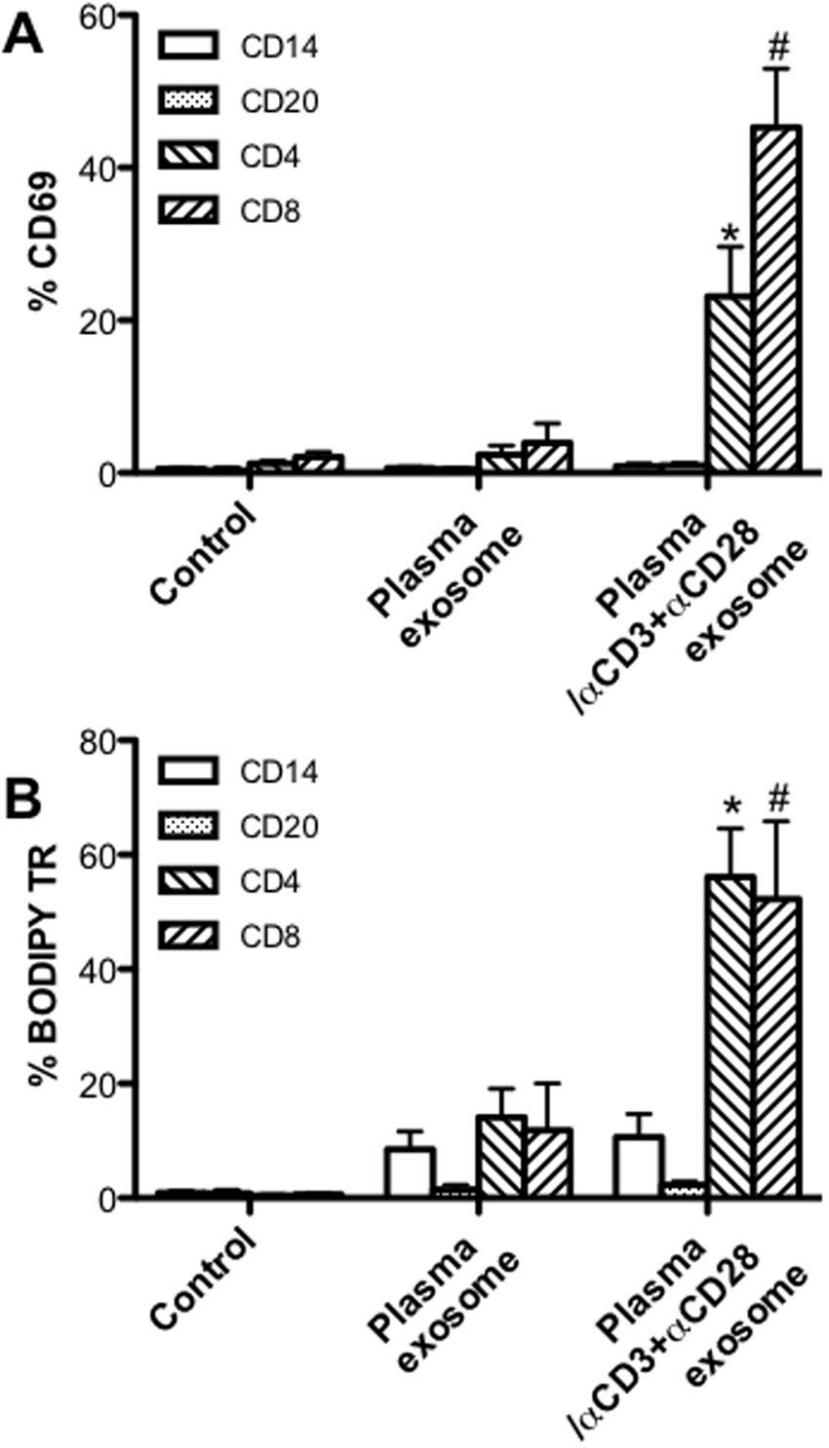
Plasma exosome uptake by peripheral blood mononuclear cells. (**A**) Activation of cell subsets after incubated with plasma exosomes, with or without TCR activation; (**B**) Percentage of BODIPY TR positive cell subsets including CD14+ monocytes, CD20+ B cells, CD4+ and CD8+ T cells with exosome uptake, with or without TCR activation. PBMCs (1 × 10^6^) were incubated with BODIPY TR-labeled plasma exosomes (10^8^) for 24 hours *in vitro* in presence or absence of anti-CD3/CD28. Error bars represent means ± SE. ^*,#^P < 0.05, compared with group without TCR-mediated activation.

To examine exosome uptake by cell subsets of peripheral mononuclear cells, the BODIPY TR-labeled plasma exosomes were incubated with PBMCs in the presence or absence of TCR activation by anti-CD3/CD28 Ab. The results showed that T cells were specifically activated by anti-CD3/CD28 via TCR signaling in the absence of feeder cells or antigen, compared with monocytes and B cells, as indicated by up-regulation of early activation marker CD69 on both CD4+ and CD8+ T cells (Fig. 2A). Exosome fusion to monocytes and B cells was observed, to some extent, whereas exosomes were significantly taken up by CD4+ and CD8+ T cells, as shown higher percentage of BODIPY TR Ceramide positive T cells, which were activated by TCR (Fig. 2B). These findings indicated that cell activation appears to enhance exosome uptake, which could have important effects on the regulation of cell function.

### Exosomes derived from IL-2 activated CD4+ T cells are preferentially internalized by autologous cells, and contribute to reactivation of resting CD4+ T cells from SIV-infected macaques

**Figure 3:**
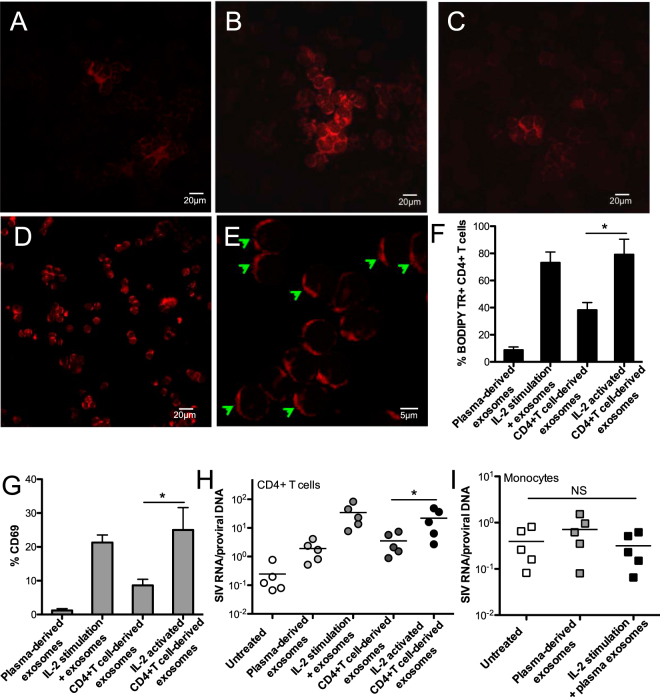
Autologous exosome uptake by CD4+ T cells, and its effects on reactivation of resting CD4+ T-cells from SIV-infected macaques. Confocal image analysis of exosome fusion to PBMCs, in which PBMCs were incubated by BODIPY TR-labeled plasma exosomes only (**A**, 20 μm), CD4+ T cell-derived exosomes in presence of IL-2 in media (**B**, 20 μm), CD4+ T cell-derived exosomes only (**C**, 20 μm), or exosomes which were isolated from supernatants of IL-2-activated CD4+ T cells (**D**, 20 μm; **E**, 5 μm); (**F**) Percentage of BODIPY TR positive CD4+ T cells that internalize BODIPY TR-labeled exosomes; (**G**) Activation of CD4+ T cells after exposure to exosomes. PBMC and plasma were isolated from SIV naïve animals, and further CD4+ T cells were isolated and cultured for exosome preparation. Effects of exosomes on viral transcription rate in resting CD4+ T cells (**H**) or monocytes (**I**) from chronically SIVmac251-infected macaques. Levels of cell-associated SIV DNA and RNA were measured after 24 hours. Significance determined by a Wilcoxon signed rank test. *P < 0.05. Error bars represent means ± SE.

It is reported that T-cell activation triggers exosome release^4,50^, and exosomes are also able to activate resting CD4+ T cells following fusion, thereby enhancing susceptibility and promoting HIV replication through various signal transduction pathways^19,22,51,52^. To investigate the efficiency of autologous exosome uptake by CD4+ T cells, as well as possible effects on resting CD4+ T-cell activation, the exosomes were isolated either from plasma or supernatants of cultured CD4+ T cells in the presence or absence of IL-2 stimulation for 24 h *in vitro*. After incubation with autologous CD4+ T cells, the results revealed that exosomes derived from IL-2-stimulated CD4+ T cells could efficiently increase capture and rapid fusion to self-CD4+ T cells, compared with plasma-derived exosomes, or those from CD4+ T cells without IL-2 stimulation (Fig. 3A–D). IL-2 can promote T cell survival, proliferation and activation, drive CD4+ T cell expansion forming T-cell colonies^53^, obviously promoting T-cell activation and counterpart exosome uptake (Fig. 3B). As indicated in Fig. 3D and E, the membrane sites where the BODIPY TR-labeled exosomes merged to CD4+ T cells displayed capping formation of a phospholipid bilayer on the CD4+ T cells. These results were consistent with the high percentage of BODIPY TR Ceramide positive CD4+ T cells after fusion to exosomes derived from IL-2-activated autologous cells (Fig. 3F). Notably, uptake of exosomes from IL-2-stimulated CD4+ T cells could markedly promote activation of CD4+ T cells, as indicated by up-regulation of CD69 expression, compared with either plasma or untreated CD4+ T cell-derived exosomes (Fig. 3G). These data indicated that exosomes released by IL-2 stimulated CD4+ T cells possess distinct effects on activation of CD4+ T cells, prompting us to further test their potential in reactivation of HIV latency. Total CD4+ T cells were purified from animals pre-infection and cultured for 24 h at 37 °C in CO2 incubator with or without IL-2 treatment, and the exosomes were isolated, quantified and kept in −70 °C for use. The resting CD4+ T cells in lymph nodes or peripheral monocytes, isolated from chronically SIV-infected macaques, were incubated with autologous exosomes. To evaluate reactivation of cells, the viral transcriptional rate in cells, i.e., the ratio of viral RNA to proviral DNA, was calculated. The results showed that exosomes derived from IL-2 activated CD4+ T cells promoted viral transcription in resting CD4+ T cells, as indicated by a significant increase in the viral RNA/DNA ratio, equivalent to IL-2-mediated T-cell activation (Fig. 3H), compared with limited effects of plasma exosomes on monocytes (Fig. 3I). These findings suggested that exosomes could be candidate vesicles to reactivate HIV latency, in combination their role as therapeutic carriers with antiretroviral therapy.

## Discussion

Exosomes are important mediators of intercellular communication in both physiological and pathophysiological states. To our knowledge, this is the first study to characterize the potential of exosomes to reactivate HIV latency in the non-human primate animal model. Our data indicate that exosomes from IL-2 stimulated CD4+ T cells can efficiently fuse and activate autologous cells, and reactivate latently SIV-infected CD4+ T cells. As both a therapeutic carrier and vesicle to reactivate HIV latency, exosomes might have a potential application in AIDS therapy.

Natural exosomes contain components that promote binding and uptake by specific target cells. Exosomes possess many unique characteristics that make them attractive candidates for *in vivo* delivery of payloads: 1) extensive production in circulation and body fluids; 2) small size with ability to deliver cargo to adjacent or distant cells, even across the blood-brain barrier (BBB); 3) lack of immunogenicity as autologous vesicles^54^, and; 4) functional self-membrane proteins and lipids, facilitating to better fusion to specific recipient cells. Additional advantageous features of exosomes include their stability, allowing them to be stored at −80 °C for over two years without loss of activity. Since they are static biochemical entities, there is no need to expand, and they can be used directly, either as stand-alone vaccines or in combination with other pharmacological agents. The concept of using exosomes for gene delivery has substantial potential in the transportation of mRNAs, siRNA, or microRNAs^16,54,55^. For therapeutic purposes, self-derived exosomes provide safe and effective strategies for cell-free payload delivery, avoiding immunogenicity of the delivery vehicle with repeated dosing or treatment. Exosomes are thus harnessed as potential therapeutic carriers, although questions still remain if this is an efficient, robust, and scalable approach to load cargo, and about target cell specificity.

Exosomes are able to traverse biological barriers and widely spread in nearby or distant tissues/organs. Further, exosomes have been implicated in normal as well as pathophysiological states, such as immune response, neuronal function, neurodegenerative diseases, cancer, and viral infections^56,57^. To investigate the cellular tropism of exosomes, the fluorescence-labeled plasma exosomes were incubated with PBMCs. Our data showed that uptake of plasma exosomes correlates with the activation status of recipient cells (Fig. 2A), as TCR activation significantly increased exosome capture by T cells (Fig. 2B), consistent with previous report that activated cells recruit exosomes^58^. The number of exosomes taken up by recipient cells likely depends on several factors, including cell type, cell activation status, and micro-environmental conditions *in vivo* ^59^.

Cell activation could trigger EV release^60^, in which exosomes display phenotype differences under conditions of oxidative stress or serum starvation^61^. For example, the status of CD4+ T cell activation determines the differential release of distinct populations of nanosized vesicles^50^. Exosomes secreted by specific T-cell subsets can modulate the activity of immune cells^11^. CD4+ T cells play the central role in adaptive and humoral immune responses and serve as major targets during HIV/SIV infection. We thus explored the correlation of exosome release and uptake by CD4+ T cells. Since nonhuman primates have similarities to humans with regard to major histocompatibility complex (MHC), we tested effects of exosomes on autologous parent cells. Our data showed that exosomes isolated from IL-2 stimulated CD4+ T cells could be markedly taken up by autologous CD4+ T cells, accompanied by increased cell activation, equivalent to IL-2 treatment (Fig. 3A–G). The reason why CD4+ T cell-derived exosomes upon IL-2 stimulation could increase capture by autologous cells remains unknown. The uptake mechanism between exosomes and target cells may depend on proteins and glycoproteins found on their surface^62^. It is speculated that proteins embed in exosome membranes, such as cell origin-associated molecular ligands, might be upregulated in these IL2-activated CD4+ T cell-derived vesicles. The exosomes from activated T cells are reported to contain T-cell receptor subunits, Src-like tyrosine kinases, adhesion molecules, and other ligands^4^, all of which are helpful for exosomes to bind their receptors on recipient cells, facilitating membrane fusion between vesicles and cells. Therefore, the cell origin of exosomes and counterpart recipient cells may determine the efficiency of membrane fusion.

HIV-1 budding and transmission from infected cells in the form of exosomes remains a controversial topic^63–65^. Although exosomes derived from DCs or macrophages in HIV+ patients could increase HIV infectivity of CD4+ T cells^66,67^, infectious HIV particles have not been directly measured. Notably, it appears that exosomes from uninfected cells are able to reactivate the quiescent CD4+ T cells once ingested^19^, enhance susceptibility, and promote HIV replication through various signal transduction pathways^51^. Molecules involved in signal transduction include Toll-like receptors (TLRs), NF-κB pathways, RNA Polymerase II, heat shock proteins (HSPs), and MHC class I and II^18–20,68^. There is precedence for viral proteins being packaged into exosomes in infected cells. Indeed, HIV proteins p24 (capsid) and Nef, and trans-activating response (non-coding TAR RNA) element have been reported to be present in exosomes that are secreted from the infected cell, thereby eliciting HIV latency reactivation and modulating cellular responses^21,22,52^. Consistent with another report^69^, our data indicated that exosomes derived from IL-2-stimulated SIV naïve CD4+ T cells could promote activation of autologous quiescent cells, even reactivation of resting CD4+ T cells from chronically SIV-infected macaques, as indicated by increased viral RNA/DNA ratio (Fig. 3H). In comparison, limited viral transcripts were measured in monocytes, which were incubated with plasma exosomes (Fig. 3I), possibly attributing to decreased frequency of SIV DNA+ monocytes at chronic stage^70^. Beside possible upregulated ligands and signaling molecules in exosomes that are isolated from IL-2 stimulated CD4+ T cells, other factors, such as viral protein and TAR from a proportion of SIV-infected CD4+ T cells that are initially activated by exosomes, might subsequently contribute to reactivation of left cells. IL-2 treatment is able to stimulate T-cell activation and induce limited proviral HIV gene expression in latently infected T cells as non-antigen immunotherapy^71,72^, however, it also leads to systemic and pleiotropic toxicity, such as imbalance of T cell subsets^73,74^. The other small-molecule LRAs, such as HDACi and PKC activators, could reactivate HIV latency to some extent, but these compounds may cause strong inflammatory responses or other potential side effects. For example, both HDACi (such as suberoylanilide hydroxamic acid, SAHA) and bryostatin-1 (a PKC activator) treatment may impair HIV-specific CTL responses^75–77^. Therefore, it is valuable to test suitable approaches for reactivation of HIV latency with low or minimal cytotoxicity, especially for their potential in HIV therapy *in vivo*. Together, autologous exosomes could induce activation of resting CD4+ T cells and reactivation of latently SIV-infected CD4+ T cells, suggesting that exosomes could have potential in HIV therapy as latency reversing vesicles and carrier in HIV+ patients on ART.

## Materials and Methods

### Ethics statement

All animals in this study were housed at the Tulane National Primate Research Center in accordance with the Association for Assessment and Accreditation of Laboratory Animal Care International standards. All studies were reviewed and approved by the Tulane University Institutional Animal Care and Use Committee. Animal housing and studies were carried out in strict accordance with the recommendations in the Guide for the Care and Use of Laboratory Animals of the National Institutes of Health (NIH, AAALAC #000594) and with the recommendations of the Weatherall report: “The use of non-human primates in research”. All clinical procedures were carried out under the direction of a laboratory animal veterinarian. All procedures were performed under anesthesia using ketamine, and all efforts were made to minimize stress, improve housing conditions, and to provide enrichment opportunities (e.g., objects to manipulate in cage, varied food supplements, foraging and task-oriented feeding methods, interaction with caregivers and research staff).

### Animals and virus

A total of 5 adult Indian-origin rhesus macaques (*Macaca mulatta;* RMs) were utilized in the study. These macaques were intravenously infected with 100TCID_50_ SIVmac251. The SIV-infected animals at the chronic stage were defined as animals infected with SIV for >3 months with no overt clinical signs of disease (chronic asymptomatic). EDTA and heparinized blood were collected from these animals at pre-infection and post SIV infection.

### Cell isolation and processing

Plasma was isolated from EDTA venous blood by density gradient centrifugation (1000 g × 20 min at RT). Peripheral mononuclear blood cells (PBMCs) from heparinized blood were prepared by with Lymphocyte Separation Medium (LSM, MP Biomedicals, Santa Ana, CA). The CD4 T cells were isolated from PBMCs by negative selection using the CD4 T+ Cell Isolation Kit (nonhuman primate) (Miltenyi Biotec, Auburn, CA). Resting CD4 T cells in chronically SIV-infected macaques were further purified by negative selection using PE-labeling anti-CD69, anti-CD25 and anti-HLA-DR antibodies and subsequent anti-PE microbeads to remove activated cells. Monocytes were isolated using PE-conjugated HLA-DR and CD14 beads. Purified CD4+ T cells (1 × 10^7^) were cultured for 24 h in 10% exosome-depleted FBS (System Biosciences) with or without 10% human IL-2 (Hemagen, Columbia, MD), and cell supernatants were collected for exosome isolation.

### Exosome isolation and quantification

Plasma or culture supernatants were centrifuged at 1000 × g for 20 minutes to remove remaining cell debris. Total exosomes were isolated with Total Exosome Isolation (Invitrogen) as described in the manufacturer’s protocols. In brief, 200 μl plasma was diluted with 100 μl 1× PBS, and 60 μl of exosome precipitation reagent was added. For media exosome isolation, exosome precipitation reagent was mixed with 1 ml cell supernatants. The mixtures were vortexed briefly and incubated at room temperature for 10 minutes. Following the incubation, samples were centrifuged at 10000 × g for 10 minutes to pellet the exosomes. Exosomes were resuspended in 200 μl 1× PBS in a 37 °C incubator for ~1 hour. Purified exosomes were quantified using EXOCET Exosome Quantification Kit (System Biosciences).

### Exosome staining

Exosomes were stained by phospholipid bilayer BODIPY® TR Ceramide (Invitrogen) or CD63 (H5C6, BD). In brief, 1 μl TR Ceramide stock solution (dissolved in DMSO, 1 mM) or 20 μl anti-CD63 was added to 3 × 10^8^ exosomes in 200 μl, incubated for 20 minutes at 37 °C. Labeled exosomes were then purified to remove free dye (or free Ab) by Exosome Spin Columns (Invitrogen), spun onto glass slides (10^8^ in 200 μl), dried overnight, and analyzed by confocal image analysis.

### Uptake of exosomes by recipient cells and cell-associated viral load determinations

To measure the uptake of exosomes, PBMCs (1 × 10^6^, 1 ml) from pre-infection were added BODIPY TR-labeled plasma exosomes (10^8^ in 50 μl) in a 24-well plate for 24 hours at 37 °C in CO2 incubator with or without stimulators (5.0 μg/mL anti-CD3 and 2.0 μg/ml anti-CD28 Ab), and the group without exosomes as the control. In other experiments, CD4+ T cells (5 × 10^5^, 1 ml) were incubated for 24 h with 10^6^ BODIPY TR-labeled autologous exosomes isolated from supernatants of IL-2 activated CD4+ T cells, exosomes from cultured CD4+ T cells, or plasma with or without IL-2 stimulator, or media alone. Monocytes were incubated with plasma-derived exosomes. The cells were harvested, washed and stained with: CD3 (SP34), CD4 (SK3), CD8 (SK1), CD14 (M5E2, BioLegend), CD20 (2H7), CD69 (FN50), and LIVE/DEAD Fixable Aqua Dead Cell Stain Kit (Invitrogen, Grand Island, NY). All antibodies and reagents were purchased from BD Biosciences Pharmingen (San Diego, CA) unless otherwise noted. Samples were resuspended in BD Stabilizing Fixative (BD Biosciences) and acquired on a FACS FORTESSA (Becton Dickinson, San Jose, CA). Data were analyzed with FlowJo software (Tree Star, Ashland, OR). To evaluate the effects of exosome uptake on reactivation of resting CD4+ T cells or monocytes, which are isolated from lymph nodes or blood in chronically SIV-infected macaques, the cells (5 × 10^5^) were incubated with 10^6^ exosomes for 24 h. Cells were then collected to analyze membrane fusion, or measure cellular viral DNA and RNA by quantitative PCR with SIV gag-specific primers^78^.

### Statistics

Statistical analyses were performed by Mann-Whitney *t* test or Wilcoxon matched-pairs signed rank test (two tailed) using GraphPad Prism 4.0 (GraphPad Software, SanDiego, CA). Significant statistic differences are indicated. Asterisks denote *p* values (*p < 0.05,). The data are presented as the mean and SE.
